# Risk of SARS-CoV-2 transmission by aerosols, the rational use of masks, and protection of healthcare workers from COVID-19

**DOI:** 10.1186/s13756-020-00763-0

**Published:** 2020-07-06

**Authors:** Rami Sommerstein, Christoph Andreas Fux, Danielle Vuichard-Gysin, Mohamed Abbas, Jonas Marschall, Carlo Balmelli, Nicolas Troillet, Stephan Harbarth, Matthias Schlegel, Andreas Widmer, Swissnoso, Carlo Balmelli, Marie-Christine Eisenring, Stephan Harbarth, Jonas Marschall, Didier Pittet, Hugo Sax, Matthias Schlegel, Alexander Schweiger, Laurence Senn, Nicolas Troillet, Andreas F. Widmer, Giorgio Zanetti

**Affiliations:** 1 Department of Infectious, Diseases and Hospital Hygiene, Freiburgstrasse, 3010 Bern, Switzerland; 2 Swissnoso, the National Center for Infection Control, Bern, Switzerland; 3 Department of Infectious Diseases and Hospital Hygiene, Aarau Cantonal Hospital, Aarau, Switzerland; 4 Department of Infectious Diseases, Thurgau Cantonal Hospital, Thurgau, Switzerland; 5 Infection Control Programme and Division of Infectious Diseases, University of Geneva Hospitals and Faculty of Medicine, Geneva, Switzerland; 6 Infection Control Programme, EOC Hospitals, Ticino, Switzerland; 7 Service of Infectious Diseases, Central Institute, Valais Hospitals, Sion, Switzerland; 8 Division of Infectious Diseases and Hospital Epidemiology, Cantonal Hospital St. Gallen, St. Gallen, Switzerland; 9 Department of Infectious Diseases, University Hospital Basel, Basel, Switzerland

**Keywords:** COVID-19, SARS-CoV-2, Aerosol, Droplet, Infection control, Transmission, Mask

## Abstract

**Objectives:**

To determine the risk of SARS-CoV-2 transmission by aerosols, to provide evidence on the rational use of masks, and to discuss additional measures important for the protection of healthcare workers from COVID-19.

**Methods:**

Literature review and expert opinion.

**Short conclusion:**

SARS-CoV-2, the pathogen causing COVID-19, is considered to be transmitted via droplets rather than aerosols, but droplets with strong directional airflow support may spread further than 2 m. High rates of COVID-19 infections in healthcare-workers (HCWs) have been reported from several countries. Respirators such as filtering face piece (FFP) 2 masks were designed to protect HCWs, while surgical masks were originally intended to protect patients (e.g., during surgery). Nevertheless, high quality standard surgical masks (type II/IIR according to European Norm EN 14683) appear to be as effective as FFP2 masks in preventing droplet-associated viral infections of HCWs as reported from influenza or SARS. So far, no head-to-head trials with these masks have been published for COVID-19. Neither mask type completely prevents transmission, which may be due to inappropriate handling and alternative transmission pathways. Therefore, compliance with a bundle of infection control measures including thorough hand hygiene is key. During high-risk procedures, both droplets and aerosols may be produced, reason why respirators are indicated for these interventions.

## Background

Regarding the optimal protection of healthcare workers (HCW), there are concerns about the use of surgical masks versus respirators (such as FFP2 or N95) when caring for COVID-19 patients in healthcare settings. There are many enquiries from healthcare institutions and HCWs as to whether surgical masks offer sufficient protection compared to respirators when providing care to a patient with COVID-19.

The aim of this article is to review the epidemiology of COVID-19 in HCWs, to interpret the scientific data on aerosol versus droplet transmission of SARS-CoV-2 (the causative agent of COVID-19), to compare basic characteristics between surgical masks and respirators, and to evaluate the evidence of protection between these two mask types. This will provide the scientific basis for the current recommendations on the use of surgical masks versus respirators for HCW in contact with COVID-19 patients.

### Epidemiology of COVID-19 in healthcare workers

Within 4 months, the COVID-19 pandemic has caused more than four million documented infections and over 300′000 deaths [1]. Work-related infections of HCWs have been described early on and provoked vivid discussions about the optimal personal protective equipment (PPE). Wu et al. reported an infection rate of 3.8% among HCWs in Wuhan [2], others reported up to 29% of HCWs being infected [3]. In the Netherlands, 86 of 1′353 HCWs (6%), all presenting with fever and/or respiratory symptoms within 1 month after the onset of the epidemic in their country, were found to be SARS-CoV-2 positive [4]. Importantly, no more than 3/86 had been exposed to an inpatient with COVID-19 infection, suggesting acquisition of the virus in the community. For the United States, the US Centers for Disease Control and Prevention (CDC) reported an average infection rate of 3% in HCW with certain states experiencing rates of up to 11%, possibly due to more complete reporting of HCW status [5]. Infected HCW indicated exclusive healthcare exposure in 55%, exclusive household exposure in 27% and exclusive community exposure in 13% [5]. In March 2020, positivity rates of HCWs in an English hospital with symptom-based screening showed an exponential increase within 20 days from 5 to 20% [6]. Remarkably, employees with and without direct patient contact showed similar incidence rates of COVID-19, implying that community-acquired disease or transmission among co-workers were more likely than nosocomial transmission from infected patients [6]. The observation that infection rates of HCWs in this study dropped as the prevalence in the hospital increased further supported the hypothesis that employees of the hospital had mainly been infected outside the healthcare setting.

The high transmissibility of coronavirus has been reported before for both, SARS-1 and MERS, with proportions of infected HCW ranging from 13 to 43%, based on country-specific data. For individual outbreaks, up to 59% of affected individuals were HCWs [7].

### Transmission pathways

COVID-19 may spread in four ways. The proposed three “direct” transmission ways are i) by infectious droplets expulsed by coughing or sneezing onto a mucous membrane (mouth, nose, eyes); ii) by aerosols from established sources such as mechanical ventilation or bronchoscopy, but - and this is controversial – may also include singing or even talking; and iii) by direct contact (e.g., by kissing, touching hands or other parts of a body contaminated with infectious respiratory or fecal material). The fourth, indirect transmission way is by contact with contaminated surfaces (fomites). However, fomites are considered likely rare sources of transmission [8–13].

The lack of understanding of the detailed mechanisms of transmission may explain the discrepancy of the recommendation to protect the HCWs with surgical masks versus respirators: on the one hand, World Health Organization, Public Health England, and Swissnoso recommend the use of surgical masks to protect against droplet transmission of SARS-CoV-2 and limit use of respirators for aerosol-generating procedures (AGPs) [8, 11, 12]. On the other hand, the United States Centers for Diseases Control and Prevention, the European Centre for Disease Prevention and Control, and the German Robert Koch Institute recommend universal use of respirators for protection against airborne transmission, where available [9, 10, 14].

### What is the evidence for SARS-CoV-2 transmission via aerosols?

#### Reproductive number, superspreading events and asymptomatic transmission

Based on i) an initial reproductive number (R_0_) of ~ 2 [15] - as compared to 18 for measles, a classical example of airborne transmission [16] - and ii) low secondary attack rates, the person-to-person spread of SARS-CoV-2 has been postulated to occur predominantly through droplet and contact transmissions. Secondary attack rates for household and close contacts (mostly defined as spending > 15 min in < 2 m distance) were 10.5 and 0.45% in the US, and 14.9 and 9.6% in Shenzhen, respectively [17, 18].

A superspreader is an individual who is more likely to infect others, compared with a typical infected person. Superspreading events have occurred during the SARS-1 and MERS epidemics and also occurs with SARS-CoV-2: Recently, one choir singer likely infected 53/61 attendees during singing practice [19]. Host, pathogen, environmental and behavioral factors may drive superspreading events, leading to an effective reproduction number similar as observed with aerosol-transmitted pathogens. But, this does not necessarily support aerosol transmission [20]: Singing in chorus may be associated with a massive expectoration of droplets. Together with the close distance between singers and poorly ventilated rooms, this may lead to a R_0_ mimicking the one of aerosol transmission. At the end, droplet versus aerosol transmission within close distances and a high inoculum is likely to be a continuum [21]. Finally, given the high transmissibility of COVID-19 in oligo- and pre-symptomatic patients, a plausible and important hypothesis is that a face-to-face conversation might be adequate to transmit COVID-19, even if both individuals do not touch each other, but this does not imply aerosol transmission [13].

#### Infectious droplets and aerosols

An infectious aerosol is a collection of pathogen-laden particles in air, usually with a diameter of below 5–10 μm [22]. Aerosol transmission occurs when infectious aerosols are generated by an infectious person, the pathogen remains viable in the air for some period of time, and the target tissues in which the pathogen initiates infection can be reached by the aerosol [22]. Particles ≤10 μm are considered respirable particles capable of reaching the lower airways, whereas particles with 10–100 μm are considered inspirable particles limited to reach the upper airways [23].

**Fig. 1:**
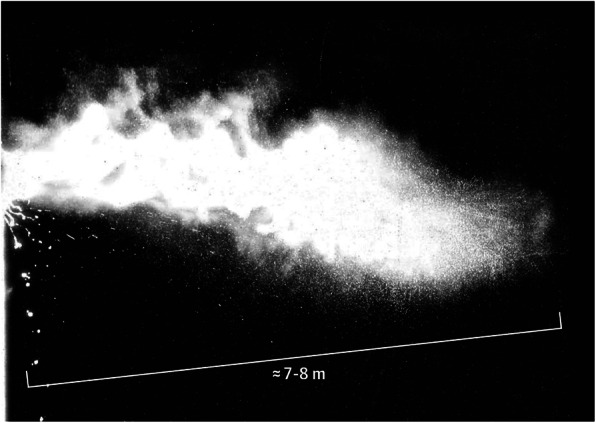
Multiphase Turbulent Gas Cloud from a Human Sneeze. Reprinted with written permission from JAMA [21]

Recent work has demonstrated that sneezing and coughing not only generates mucosalivary droplets, but also a multiphase turbulent gas (a puff) with a highly diverse and volatile composition that can span 7–8 m (Fig. 1) [21]. Of note, the impact of this finding on infections is yet unknown. Large droplets may settle faster than they evaporate, contaminating the immediate vicinity of the infected individual. Alternatively, the moist and warm atmosphere within the turbulent gas cloud may prevent evaporation for much longer than occurs with isolated droplets, thus mimicking aerosol transmission. In contrast, small droplets may evaporate faster than they settle forming true “droplet nuclei” or aerosols.

Most knowledge on aerosols has been derived from experimental machine-generated particles, which makes extrapolation to the clinical setting difficult. Aerosols with ≤10% of the particles being > 8 μm may be generated by breathing, talking or singing [13, 24], while in natural coughing 99% of the particles are larger than 8 μm and therefore mostly represent droplets [25].

#### Clinical evidence for true aerosol transmission of SARS-CoV-2 in humans?

A recent laboratory study showed that artificially aerosolized SARS-CoV-2 survived in the air as long as SARS-CoV-1 and persisted even longer on surfaces, from where it might become resuspended by turbulent air [26]. However, these in vitro results are not consistent with the observation of R_0_ around 2, and the rapid decrease of the incidence of SARS-CoV-2 after limiting socializing in Switzerland to less than 5 individuals [15].

Several clinical studies have identified SARS-CoV-2 RNA in air samples and significant environmental contamination, yet without documentation of viable virus [27–30]. This indicates that SARS-CoV-2 is shed to the environment as small, virus-laden particles, during toileting, through contact with fomites and from infected patients, but again this does not prove that it corresponds to infectious aerosols [27–29]. Accordingly, airflow can disperse particles with viral RNA in a room; however, this does not prove COVID-19 to be a truly aerosol-transmitted disease.

In contrast, transmittable virus had been cultured from fomite samples in a MERS-CoV outbreak in South Korea [31]. Such data have resulted in guidelines unanimously requiring contact isolation in addition to droplet precautions and propagating strict hand hygiene.

Beyond SARS-CoV-2, patients with influenza, human corona- and rhinovirus infections shed small aerosol particles < 5 μm in exhaled breath [32]. For influenza, viable virus could be cultured from such aerosols [24]. Yan and coworkers isolated infectious virus in 39% of fine aerosols collected from breath of influenza patients, yet with a concentration 4 logs lower than in nasopharyngeal swabs, which may question transmissibility in a clinical context [33]. Fine-particle exhaled aerosols (<5um) reflected infection in the lung; sneezing and coughing were not necessary for infectious aerosol generation. The conclusion was that influenza infection in the upper and lower airways is compartmentalized and as such behaving independently [33].

Despite these data demonstrating contagious aerosols of influenza in an experimental context, its transmission has successfully been prevented with droplet precautions in clinical practice, casting doubt on the generalizability of these experimental laboratory based findings in the clinical setting.

Finally, viable SARS-1 has not been detected in aerosols emitted by or generated from an infectious person. Experimental infection in SARS-1 has never been demonstrated via the aerosol route [22].

#### SARS-CoV-2 transmitted by droplets with a range > 2 m

**Fig. 2:**
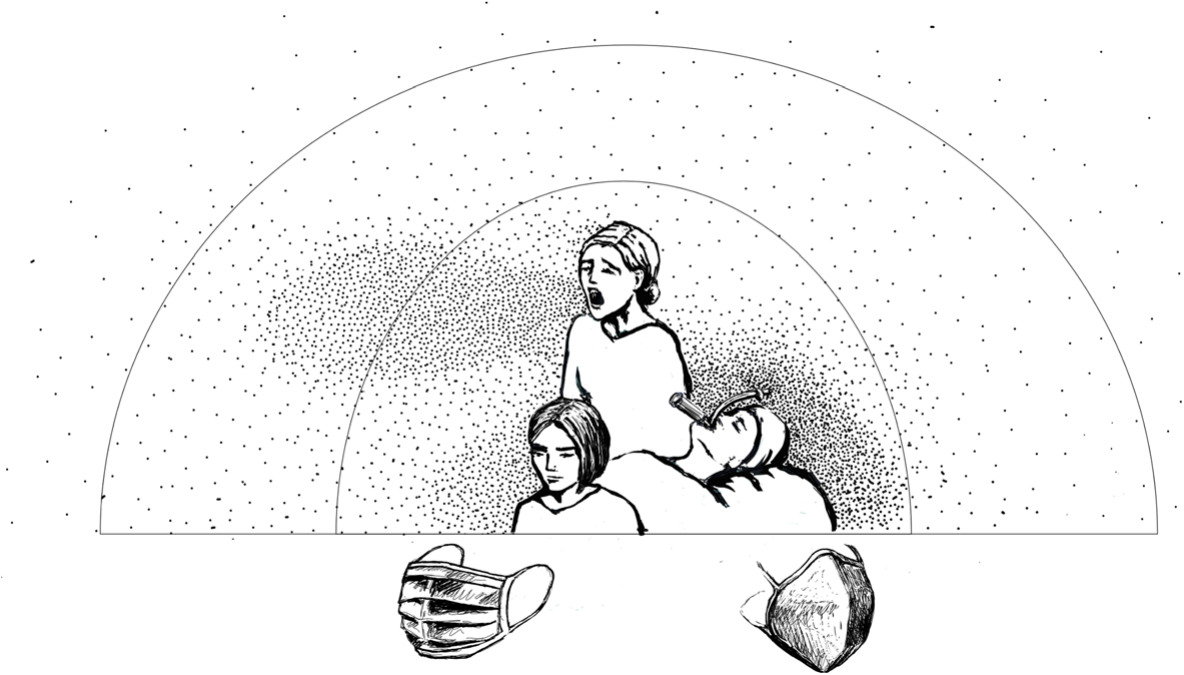
Droplet transmission and high-risk procedures (potentially generating aerosol). Inner/outer semicircle indicate 2/8 m distance from the patients (center). Center-Right: A high-risk transmission procedure is depicted (“potentially aerosol generating procedure”), where a FFP2 mask is required. Center-Left: Uncontrolled coughing in hospital may cause a turbulent gas cloud to spread beyond 2 m [21]. Regular speech, even in asymptomatically infected patients may generate infectious droplets that travel 1-2 m. This is the rational of HCW to wear surgical masks in the hospital when caring for patients

Based on the above, transmission of respiratory infections cannot be dichotomized in classical droplet transmission within a range < 2 m and aerosol transmission beyond 2 m distance (Fig. 2). Rather, more recent studies describe droplets to travel in the air for > 2 and up to 8 m - long enough to not fall under the common classification of “droplet” infection [19, 21, 34]. The postulated droplet range < 1-2 m is largely based on a publication from 1942 using still photography [35] and from work of Hall et al. on RSV from 1977 to 1982 [36]. However, in contrast to aerosols, viable droplets will ultimately fall to the floor, and will neither stay airborne nor infectious for several hours like a typical aerosol transmitted virus such as measles or varicella virus would.

In January 2020, a COVID-19 outbreak occurred in an air-conditioned restaurant in Guangzhou, China. It involved three family clusters > 2 m apart, suggesting aerosol transmission. However, when considering airflow direction and air exchange rates, findings were compatible with droplet transmission [37].

A SARS-1 outbreak in airline passengers was related to the physical proximity to the index patient, with illness reported in 8 of the 23 persons who were seated in the three rows *in front* of the index patient, as compared with 10 of the 88 persons who were seated elsewhere [38]. Directed air ventilation or strong unobstructed coughing/sneezing by the index patient may have supported the transmission to exposed passengers primarily in the front [38].

As a consequence, social distancing by ≥2 m may not always be enough to prevent droplet infections in a setting with uncontrolled coughing, sneezing and turbulent air flow such as in hospitals. Surgical masks for HCWs, as currently practiced in most Swiss hospitals, seems prudent in such settings [12]. This is further supported by recent work demonstrating how surgical masks prevent the emission of viable viral particles [39]. Further studies which illuminate the narrow line between droplet and aerosol are clearly needed [34].

#### “Aerosol-generating procedures” (AGP)

This term is frequently used for procedures reported to represent an increased risk of transmission to HCW during the SARS-1 epidemic. Higher infection rates have been well documented for manual ventilation before intubation, intubation itself, tracheotomy and non-invasive ventilation (Table 1) [40]. Remarkably, it has *not* been proven that increased transmissibility in these settings is related to aerosol transmission. In the case of non-invasive ventilation, the high infection rate may also have been related to poor adherence to standard precautions [40].

**Table 1:** Procedures associated with increased risk of SARS-1 transmission to healthcare workers

Procedure	Point or Pooled estimate (OR, 95% CI)
Tracheal intubation (4 cohort studies & 4 case-control studies)	6.6 (2.3–18.9) & 6.6 (4.1–10.6)
Non-invasive ventilation (2 cohort studies)	3.1 (1.4–6.8)
Tracheotomy (1 case-control study)	4.2 (1.5–11.5)
Manual ventilation before intubation (1 cohort study)	2.8 (1.3–6.4)

Adapted from Tran et al. [40]: Risk of transmission of acute respiratory infections to healthcare workers caring for patients undergoing risk procedures compared with the risk of transmission to healthcare workers caring for patients not undergoing risk procedures. Most studies included in the systemic review assessed whether healthcare workers had proper infection control training or wore personal protective equipment while caring for patients with laboratory-confirmed SARS

A study that objectified aerosol particle generation during patient care activities only could identify nebulized medication administration as a significant source of aerosols [41]. Of importance, this work was performed in patients without viral infections and the impact on infection transmission is unknown [41]. Subsequent attempts also failed to correlate several presumable AGPs to a statistically significant probability of sampling a H1N1-RNA positive aerosol [42].

Still, several national and international guidelines stated that these presumable AGPs are high-risk procedures necessitating FFP2 protection. However, this possibly relates to high level of viral exposure from droplet clouds rather than indication transmission by the airborne route (Fig. 2) [12].

### Mask types to protect HCW from COVID-19 transmission

#### Surgical masks versus respirators

In light of this evidence, we address the question which type of mask ought to be used by HCWs to protect themselves and if this choice should be guided by individual transmission risks of specific procedures. Two main forms of disposable masks are currently being used in the Swiss healthcare system (Table 2): A standard surgical mask (type II) which forms a barrier against droplets and, optionally, splashes (type IIR). Surgical masks are intended to protect the patient (e.g., during surgery from respiratory tract bacteria exhaled by the surgical team) and undergo standardized testing before being marketed in Europe (Standard EN 14683). From practical, yearlong experience, we know though, they also protect the HCW. Of note, the size of SARS-CoV-2 is approximately 120 nm, and therefore, 10 to 50 times smaller than *Staphylococcus aureus*, the test pathogen for the effectiveness of surgical masks. Nevertheless, the virus is on droplets and therefore the size of the virus does not play a critical role.

**Table 2:** Surgical masks versus FFP2, specification according to EN standard

Certification/ Class (Standard)	FFP2 (EN 149)	Type II Surgical Mask (EN 14683)
Protection	Protection of the carrier against solid and liquid aerosols	Protection against droplet ejection from the carrier*
Application	Self protection / Industrial safety	External protection*
Filter performance – (must be ≥ X% efficient)	0.3 Microns ≥94%	3.0 Microns: ≥ 98% 0.1 Microns: No requirement
Total inward leakage	≤ 8% leakage (arithmetic mean)	No requirement
Exposure to of inert particles and live aerosolised influenza virus	Estimated 100-fold reduction**	Estimated 6-fold reduction**

*This description meets the requirements of the EN standard. However, the evidence described in the body of the article and many years of experience show that surgical masks provide sufficient self-protection

**According to the Health and Safety Laboratory for the Health and Safety Executive 2008, https://www.hse.gov.uk/research/rrpdf/rr619.pdf

Surgical masks adapt rather loosely to the face of the user and can be worn for a maximum of 8 h, but should be changed earlier when damaged or visibly wet. To avoid self-contamination, they should not be worn around the neck in-between use.

In contrast, respirators are available in Europe as FFP ("filtering face piece) masks (Standard EN 149), in the US as an N95 mask. There are three categories depending on filter performance of particles > 0.3 μm: FFP1 (> 80%), FFP2 (> 94%) and FFP3 (> 99%). FFP2 masks are intended to protect the carrier from the inhalation of airborne particles. The FFP2 testing procedure requires also a maximal level of microbial contaminated air that leaks through a respirator. This is the reason why they require fit-testing to ensure a tight seal around the user’s face. Many users perceive this as discomfort, which may interfere with compliance [43]. Of importance, FFP2 masks with expiratory valves *are not indicated* in the COVID-19 setting, as they do not protect others.

Guidelines recommend the use of surgical masks for use in suspected or confirmed COVID-19 patients, with enhanced protection using FFP2 for the so called AGPs [8, 11, 12].

#### Indirect evidence consistently supports the use of surgical masks

Most of the research comparing the protective effect of surgical masks with respirator masks has been generated from influenza or other relatively benign respiratory diseases. To date, there are no published head-to-head studies in COVID-19. The current guidelines are therefore extrapolated from influenza and previous outbreaks of SARS-1 and MERS, and on expert opinion [12, 44].

Despite the documentation of infectious influenza aerosols in clinical samples [24], evidence of aerosol transmission in clinical practice has neither been found for influenza nor for SARS. A recent meta-analysis of 4 RCTs including 6418 patients did not provide any evidence that medical masks are inferior to N95 respirators for protecting healthcare workers against laboratory-confirmed viral infection (OR 1.06, 95% CI 0.90–1.25) or influenza (OR 0.94, 0.79–1.20) [45]. In addition, a meta-analysis of observational studies provided evidence of a similar protective effect of surgical masks (OR = 0.13; 95% CI: 0.03–0.62) and respirators (OR = 0.12; 95% CI: 0.06–0.26) compared with no mask against SARS-1 [44]. Consistent results also were provided by two case-control studies in influenza and SARS-1 [46].

These findings are supported by preliminary epidemiological data from an anecdotal report describing no SARS-CoV-2 transmission in 35 HCW protected by surgical masks who were exposed to aerosol-generating procedures including intubation, extubation and non-invasive ventilation [47].

A recently published meta-analysis comparing different types of masks versus no mask reported better protection against MERS, SARS and COVID-19 in studies using FFP2 (96% protection rate) compared to surgical masks (77% protection rate) [48]. This conclusion should be seen with caution, as they are based on in-between study rather than within-study comparisons. Furthermore, studies in a healthcare setting, which more frequently used FFP2 masks, have been shown to generally perform better than studies performed in the community. Direct comparisons between the two mask types are mandatory.

In summary, the current knowledge provides no scientific evidence from head-to-head studies in favor of using FFP2 instead of a surgical mask outside the so-called AGPs.

#### When mask protection seems to fail

Independent of the respiratory precautions taken, nosocomial transmissions of respiratory viral infections may occur. This underlines that masks are only one component of complex measures that include goggles or face shields and gowns. A recent metaanalysis has described a 78% reduction in infections when eye protection was used [48]. Even more important are behavioral measures to support proper wearing (donning) and removal (doffing) as well as general infection control measures, in particular hand hygiene [49]. Particular care should be taken to ensure that masks are not contaminated on inanimate surfaces [50].

A SARS-1 outbreak among HCW in Toronto has been related to lack of adequate infection control training, inconsistent PPE use and fatigue rather than the choice of a wrong mask. More than 50% reported remembering breaches in infection control precautions [51].

Four publications have shown that a similar proportion of HCWs wearing respirators and masks became infected with influenza within the same study (Table 3). The absolute incidence of infections varied considerably across studies, stressing the importance of factors other than respiratory protection.

**Table 3:** Absolute risk of laboratory-confirmed influenza infection in healthcare workers by the type of mask worn

Study	Included Patients (n)	Randomization	Incidence of laboratory-confirmed influenza infection	
			Mask	Respirator
Loeb, 2009 [52]	446	Individual	22.2%	21.7%
MacIntyre, 2011 [53]	1441	Cluster	1.2%	0.4%
MacIntyre, 2013 [43]	1669	Cluster	0.3%	0.5%
Radanovich, 2019 [54]	2862	Cluster	7.3%	8.3%

Adapted from Bartasko et al. [45]

While masks protect HCWs from viral infections, they may not always be protective in community settings [55]. This most probably is due to incorrect donning and doffing of previously contaminated masks and lack of hand hygiene. Mask contamination occurs both by respiratory droplets from close contacts of individuals wearing the mask or by the wearer’s own hands. Viral contamination of most touchable surfaces with virus has been documented even in rooms of fully recovered patients with MERS-CoV [29, 31]. Studies recovering viable influenza virus from masks and respirators illustrate that virus trapped on the surfaces poses an indirect contact transmission risk [56]. Observations in public areas have revealed that individuals touch environmental surfaces and their mouth/nose mucosa more than 3 times per hour [57]. Considering one’s need for mask repositioning or rubbing, the rate of touching a mask presumably is even higher. When manipulated incorrectly, masks may not efficiently prevent from infection. Consistent with this hypothesis of secondary infection from a contaminated mask by hands then touching the mucosa is the observation by Aiello et al. and Cowling et al. that masks only were protective when combined with hand disinfection [58, 59].

Missed diagnoses of COVID-19 infection with a lack of adequate infection control measures represents another potential reason for protection failures. For MERS-CoV, SARS-1 and SARS-CoV-2, nosocomial transmission related to delayed diagnosis rather than the choice of the type of mask for respiratory isolation have been reported [60, 61].

## Conclusion

HCWs at the frontline have significant exposure to SARS-CoV-2 during work. In addition, infected HCW may further transmit COVID-19 to patients if PPE is not worn correctly or adherence to hand hygiene is low. The absence of clear scientific evidence for aerosol transmission of SARS-CoV-2 provide the rationale for the current recommendations for the use of surgical masks. Respirators are suggested for certain defined procedures with higher transmission risk. Importantly, masking is only one component of the infection control bundle including hand hygiene. Current data provide sufficient evidence for protection of HCW to patients and self by surgical masks.

## Data Availability

Not available (review article).
